# The Influence of Circadian Rhythm on Cancer Cells Targeting and Transfection Efficiency of a Polycation-Drug/Gene Delivery Vector

**DOI:** 10.3390/polym14040681

**Published:** 2022-02-10

**Authors:** Tânia Albuquerque, Ana R. Neves, Telma Quintela, Diana Costa

**Affiliations:** 1 CICS-UBI-Health Sciences Research Centre, Universidade da Beira Interior, Avenida Infante D. Henrique, 6200-506 Covilhã, Portugal; tania.albuquerque@ubi.pt (T.A.); ana.neves@ubi.pt (A.R.N.); tquintela@fcsaude.ubi.pt (T.Q.); 2 UDI-IPG-Unidade de Investigação para o Desenvolvimento do Interior, Instituto Politécnico da Guarda, 6300-559 Guarda, Portugal

**Keywords:** cancer-cells targeting, cancer chronotherapy, circadian clock, drug/gene nanodelivery systems, gene therapy

## Abstract

The conception of novel anticancer delivery systems and the combination of chronobiology with nanotechnology may provide a powerful tool to optimize cancer therapy. In this work, polyethylenimine (PEI) has been used to complex p53 encoded plasmid DNA (pDNA), and the anticancer drug methotrexate (MTX) has also been loaded into the vectors. To investigate the influence of circadian clock on drug/gene delivery efficiency, HeLa, C33A and fibroblast cells have been transfected with developed PEI/pDNA/MTX delivery vectors at six different time points. Phenomena as the cellular uptake/internalization, drug/gene delivery and p53 protein production have been evaluated. The cell-associated MTX fluorescence have been monitored, and p53 protein levels quantified. In HeLa and C33A cancer cells, significant levels of MTX were found for T8 and T12. For these time points, a high amount of p53 protein was quantified. Confocal microscopy images showed successful HeLa cell’s uptake of PEI/pDNA/MTX particles, at T8. In comparison, poor levels of MTX and p53 protein were found in fibroblasts; nevertheless, results indicated rhythmicity. Data demonstrate the influence of circadian rhythm on both cancer-cells targeting ability and transfection performance of PEI/pDNA/MTX carriers and seemed to provide the optimum time for drug/gene delivery. This report adds a great contribution to the field of cancer chronobiology, highlighting the relationship between circadian rhythm and nanodelivery systems, and charting the path for further research on a, yet, poorly explored but promising topic.

## 1. Introduction

Almost every living organism on Earth has developed, through evolution, a timekeeping system responsible for the generation and regulation of circadian rhythms to help these organisms cope with environmental daily changes such as light and temperature [1]. In mammals, circadian rhythms are in part controlled by a master clock located in suprachiasmatic nuclei that coordinates other peripheral clocks. At the molecular level, this central network consists of a set of circadian clock-regulated genes that interact between them, forming transcriptional autoregulatory feedback loops. As an output pathway are a range of crucial biological processes that modulate behavior patterns, physiology, and even individual cell function [2]. The coordination between all internal clocks and alignment with external cues are essential for cellular homeostasis and health.

Circadian rhythms might be vulnerable to dysfunction, either triggered genetically, through mutations in clock genes that impact their function or by environmental factors including night-shift work, jet lag, exposure to artificial light at night, feeding schedules and socializing habits [3,4,5]. Accordingly, it is not surprising the emerging evidence concerning circadian rhythms contribution to the pathogenesis process of many long-term diseases, including cancer [6,7,8]. It has been reported that several physiological functions related to cancer cellular processes, such as metabolism, cell proliferation, cell cycle, DNA damage and apoptosis are under circadian control. Therefore, changes in clock gene’s function and expression might lead to the activation of intracellular inflammatory and oncogenic signaling pathways [9,10]. Besides, the expression of p53 tumor suppressor gene is under the regulation of the circadian clock. In line with this, disruption of circadian regulation could possibly down-regulate p53 protein expression, interfering indirectly in tumor development [11]. Previous research has provided ample support for this association and shows that the activity and expression levels of p53 are directly regulated by the core-clock gene Period2 (Per2) and are reduced in Per2-deficient mice. On the other hand, p53 can influence the mRNA and protein levels of PER2, antagonizing PER2 expression by directly binding to PER2 promoter and blocking CLOCK-BMAL1 transactivation of the gene [11,12]. This bidirectional regulation is even more intricate as PER2 protein can form a dimer with p53, facilitating its translocation to the nucleus [13]. Additionally, circadian rhythms can also affect mechanisms involved in the efficacy of cancer therapy. Processes such as drug absorption, transport, distribution, metabolism, excretion, and drug bioavailability present diurnal fluctuations and are under the control of circadian machinery, ultimately modulating drug efficacy and induced toxicity [14,15,16]. These findings provide an opportunity for chronotherapy, a treatment scheduling approach, aiming improve treatment efficacy of many types of cancer and increase patient survival, while diminishing side effects and associated costs for cancer therapy [17,18]. 

Gene therapy is an attractive research field and emerges as one of the most promising tools for cancer therapy [19]. Most of the accomplished advances include strategies focused in the delivery of tumor suppressor genes, as p53, that are involved in cellular pathways such as DNA repair, regulation of the cell cycle and apoptosis [20]. p53 is responsible for keeping the genome integrity and mutations in this gene are found in more than 50% of human tumors. In comparison to conventional approaches, gene therapy allows for controlled gene release, reducing the need for multiple interventions and promotes efficient release of therapeutic genes to the tumor tissue, decreasing toxicity to healthy cells [21]. Moreover, gene therapy can be used alone or as an adjuvant to chemotherapy, since certain genes can sensitize tumor cells to drugs enhancing therapeutic outcomes by synergistic effect [22,23,24,25]. 

Despite the latest progress in the design and conception of delivery vectors, some relevant issues remain poorly studied. Research on delivery systems for cancer therapy has totally failed to address the involvement of the circadian clock in processes such as the cellular uptake of the delivery system, its release profile and therapeutic effect. The combination of chronobiology with nanotechnology is expected to overcome some limitations, enhancing therapy efficacy, and reducing side effects, towards a more personalized medicine approach [26]. In this context, Hu and co-workers [27] examined the anti-tumor effect of paclitaxel (PTX)-loaded polymeric nanoparticles combined with circadian chronomodulated chemotherapy for lung cancer therapy. This team intended to screen out the best time of the day for PTX administration. The anticancer effect displayed by developed PTX-nanoparticles was evaluated by monitoring tumor growth inhibition rate, micro-vessel density, cell proliferation and cell apoptosis, following either injection with PTX or administration of PTX-loaded nanoparticles [27]. The obtained data indicated that PTX-nanoparticles exhibited greater anti-tumor activity against A549 cells, in comparison with PTX injection, and the anti-tumor effect was more effective at a specific time administration. The reported information by Hu et al. was extremely relevant and opened the path for further research on the conception of chronomodulated drug delivery systems for progresses in cancer therapy. 

From this knowledge, our team went deep into the study of the influence of circadian clock on delivery systems by considering not only co-delivery (drug/gene) systems but also their effect on different cell lines (cancer cells, HeLa and C33A, and fibroblasts). In this work the effect of circadian clock on a set of different mechanisms regarding cancer gene therapy mediated by a drug/gene delivery system, on different cell lines, was investigated. For that purpose, the anticancer drug MTX (methotrexate) has been loaded into polyplexes based on PEI (polyethylenimine) and a p53 encoding plasmid DNA, as described before [25], and transfection was carried out at different time points. This work highlights, for the first time, the influence of circadian rhythm on the performance of co-delivery systems, intending to provide the best time for drug/gene administration in view of achieving optimal therapeutic effect.

## 2. Materials and Methods

### 2.1. Materials

Commercial branched PEI with average Mw 25 kDa, MTX hydrate, DMEM/Nutrient Mixture F-12 Ham (DMEM/F-12) with L-glutamine cell culture medium and fluorescein isothiocyanate (FITC) were all obtained from Sigma-Aldrich (St. Louis, MO, USA). DAPI was from Invitrogen (Carlsbad, CA, USA). All chemicals were of analytical grade. The 6.07 kbp plasmid pcDNA3-FLAG-p53 (Addgene plasmid 10838, Cambridge, MA, USA) used in the experiments was produced and purified by a procedure developed by our research team and described in the literature [25].

All solutions were freshly prepared by using ultra-pure grade water, purified with a Milli-Q system from Millipore (Billerica, MA, USA). Cancer HeLa and C33A cells were purchased from Invitrogen and fibroblast cells from PromoCell (Heidelberg, Germany).

### 2.2. Nanoparticle Formulation

PEI/pDNA/MTX complexes were prepared by a co-precipitation method, as described elsewhere [25]. Briefly, nanoparticles were formed by adding a solution of PEI (100 µL) to μg of pDNA (400 µL) at a nitrogen to phosphate groups (N/P) ratio of 5 with or without the addition of MTX (1.2 μg). The mixture was vortexed for 60 s and left for equilibration for 15 min at room temperature. The complexes were then centrifuged at 10,000 rpm for 20 min before use.

### 2.3. Cell Culture and In Vitro Transfection Studies

For transfection studies, HeLa, C33A and fibroblast cells were seeded at a density of 1.2 × 10^5^ cells/3.8 cm^2^ and grown in Dulbecco’s Modified Eagle’s Medium (DMEM)/F-12 with L-glutamine supplemented with 10% heat inactivated fetal bovine serum and 1% streptomycin/penicillin solution and were maintained at 37 °C in a humidified atmosphere containing 5% CO_2_. Twelve h before transfection, the complete medium was replaced by medium supplemented with 10% FBS and without antibiotics, to promote transfection. On the 3rd day of culture, cells were synchronized with 0.1 µM of dexamethasone for 2 h. Afterwards, the medium was replaced by fresh, dexamethasone free medium. In vitro synchronized Hela cells exhibit rhythmic oscillations of bmal1 expression over a 24 h cycle (Figure S1, available in the Supplementary Materials). After confirmation of cell synchronization, transfection studies were performed.

**Figure 1:**
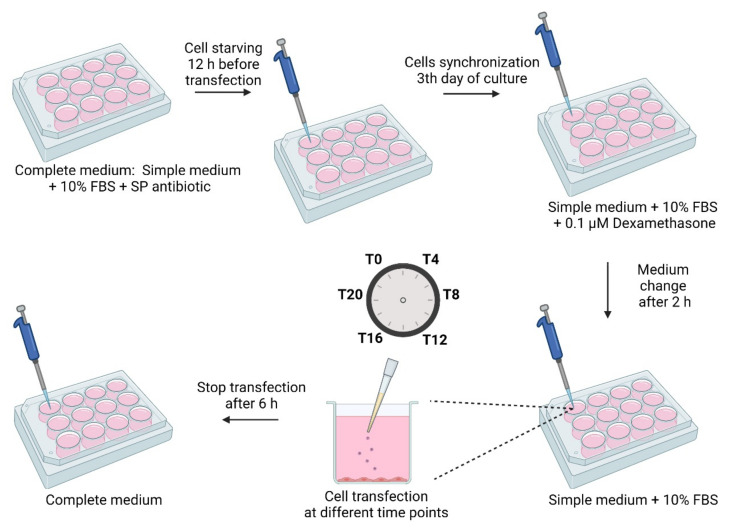
Schematic representation of the synchronization of cells and the transfection procedure.

The first time point of transfection, mediated by the nanoplexes or free MTX, was performed after cells synchronization and every 4 h through a cycle of 24 h. The transfection occurred for 6 h and subsequently cells were returned to their usual culture condition and collected at different time points. Figure 1 summarizes the experimental procedure.

### 2.4. Quantification of Cell-Internalized MTX

To evaluate if the cellular uptake of MTX is regulated in a circadian manner, cells were incubated with free MTX or with PEI/pDNA/MTX complexes. Non-transfected cells treated similarly were used as control. After 6 h of transfection, cells were washed with PBS and collected at different time points, with intervals of 4 h (T0; T4; T8; T12; T16 and T20), T0 being the time of the first collection (8 h after synchronization). Then, cells were lysed by incubation with a Triton X-100 1% solution for 30 min at 37 °C and pipetted into a black plate. Lastly, MTX concentration was determined by fluorescence reading using a SpectraMax Gemini spectrofluorometer (Molecular Devices, Sunnyvale, CA, USA) at excitation and emission wavelengths of 490 nm and 520 nm, respectively. All the experiments were performed in triplicate.

### 2.5. Fluorescence Confocal Microscopy

#### 2.5.1. FITC Plasmid Labelling

pDNA was stained with FITC by mixing 8 μg of pDNA, 75 μL of labeling buffer (0.1 M sodium tetraborate, pH 8.5) and 4 μL of FITC (in sterile anhydrous dimethyl sulfoxide, 500 mg/mL). Samples were placed under constant stirring for 4 h at 4 °C and protected from light. To stop the reaction, one volume of 3 M NaCl (85 μL) and 2.5 volumes of 100% ethanol (212.5 μL) were added. Subsequently, samples were incubated at −20 °C overnight. Thereafter, the samples were centrifuged at 4 °C for 30 min and the pellet was washed with 75% ethanol.

#### 2.5.2. Cell Live Imaging

The cellular uptake in HeLa cells was evaluated by confocal laser scanning microscopy (CLSM). HeLa cells were grown in μ-slide 8 well until 50–60% confluence was achieved. FITC-labeled pDNA alone or along with MTX was complexed with PEI to form the delivery systems. The formed complexes were visualized 6 h after transfection occurred at different time points. To stain the nucleus, cells were incubated with 1 μM DAPI for 10 min. Real live transfection was visualized using LSM 710 confocal microscope (Carl Zeiss SMT, Inc., Oberkochen, Germany) under 63× magnification and analyzed with the LSM. During the experiment, HeLa cells were maintained at 37 °C with 5% CO_2_. All images were acquired with the laser and the filters corresponding to the respective DAPI (445/450 nm) and FITC (525/550 nm) dyes.

### 2.6. Protein Quantification

The p53 protein levels have been determined by using p53 pan ELISA kit (Roche Applied Science, Basel, Switzerland), following the procedure described by the manufacturer. The protein concentration was determined by spectrophotometrically measuring the absorbance at 450 nm using a UV-Vis 1700 spectrophotometer (Shimadzu, Tokyo, Japan). All the experiments were repeated three times in triplicate. 

### 2.7. Statistical Analysis

Normality tests (D´Agostino & Pearson omnibus and Kolmogorov–Smirnov) were applied to determine the normality of distribution of the sample data. One-way analysis of variance (ANOVA) followed by Bonferroni test was used for comparing data of control and multiple experimental groups. A confidence interval of 95% (*p* < 0.05) was considered statistically significant. Data were compared and expressed as mean ± SEM. Data analysis was performed in GraphPad Prism v.8.01 (GraphPad Software Inc., San Diego, CA, USA). 

CircWave v1.4 analyses software (Dr. Roelof A. Hut, http://www.euclock.org accessed on 28 December 2021) was used to analyze circadian oscillation patterns of MTX and p53 protein quantification on HeLa cells, by harmonic regression method with an assumed period of 24 h and with α set at 0.05. The P values reported are the result of F test from software.

## 3. Results and Discussion

### 3.1. The Properties of Polyplexes

**Figure 2:**
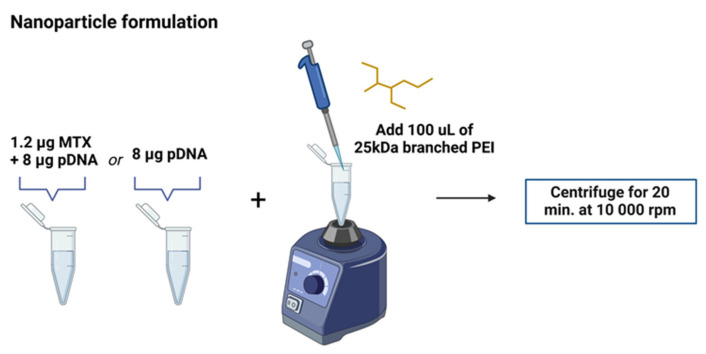
Schematic illustration of the formation of PEI/pDNA or PEI/pDNA/MTX nanoparticles following a co-precipitation method. The detailed description is available in the experimental section.

PEI is a cationic polymer existing as a branched or linear structure, known for its great ability to condense nucleic acids. Branched PEI contains primary, secondary and tertiary amine groups. Due to its high charge density PEI strongly interacts, via electrostatic forces, with negatively charged pDNA what results in the formation of PEI/pDNA complexes. Figure 2 illustrates the formation of PEI/pDNA or PEI/pDNA/MTX nanoparticles by a co-precipitation method. N/P ratio revealed to be a tailoring parameter of complexes physicochemical properties, such as size and surface charge [23]. PEI/pDNA/MTX complexes have been previously formulated and characterized by our research group [25]. The formed PEI/pDNA/MTX nanosystems (N/P ratio of 5) presented a spherical geometry, revealed by scanning electron microscopy analysis. Moreover, from dynamic light scattering, it was found that PEI/pDNA/MTX complexes exhibit lower size (155 ± 4.8) and positive surface charges (+35.1 ± 0.9). Polydispersity index (PdI) was also analyzed as a measure of size distribution. A mean PdI value of 0.3 was found for PEI/pDNA/MTX nanoparticles at N/P of 5, indicating that these nanoparticles are monodisperse. Furthermore, it was found that these nanoparticles remain stable and keep their physicochemical properties over a period of, at least, one month. Concerning payload loading/complexation ability displayed by PEI/pDNA/MTX complexes, a previous study by our team revealed high MTX and pDNA loading/encapsulation capacity [25]. 

Furthermore, in the previous work, transfection of HeLa cells mediated by these PEI/pDNA/MTX nanoparticles demonstrated the great potential of the nanocomplexes to inhibit cells viability and proliferation [25] The incorporation of MTX into PEI/pDNA complexes revealed to be a valuable tool to promote cancer cells targeting, therefore, enhancing cellular internalization, transfection and, consequently, the anticancer effect displayed by developed nanosystems [25]. From this knowledge, we found relevant to investigate these PEI/pDNA/MTX complexes within the scope of the present work.

### 3.2. The Role of Circadian Rhythms on Cellular Uptake/Internalization

The anticancer drug MTX is known to easily enter into cells through folate receptor-mediated endocytosis [28]. Once inside the cell, the phenomenon of endosomes acidification is the main source of elimination or degradation of most biomolecules. This obstacle can, however, be circumvented by the encapsulation of payloads into delivery systems, namely, PEI-based vectors. PEI presents a high charge density and chain flexibility along with a great pH buffering capacity [24,29]. Its “proton sponge effect” confers to PEI delivery systems the ability to avoid endosomes acidification and allows the release of loaded molecules into the cytoplasm [24,25,30].

**Figure 3:**
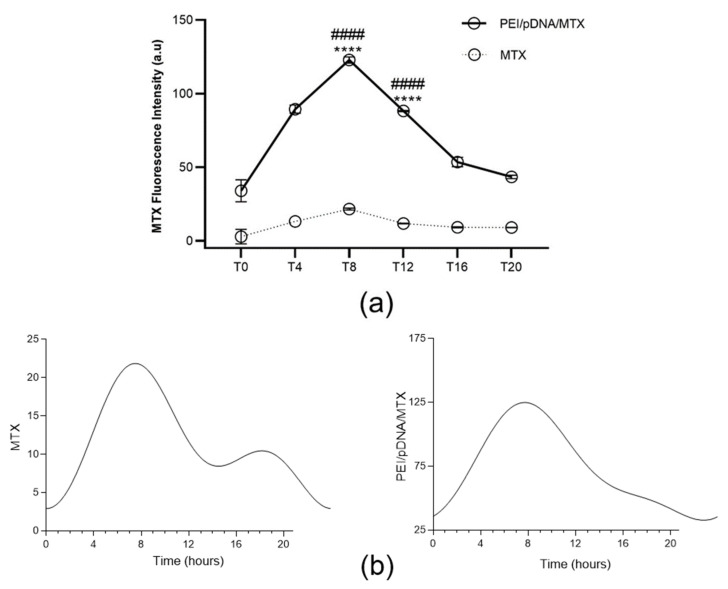
Quantification of cell-associated MTX fluorescence for each time point. (**a**) The fluorescence intensity of MTX was measured in Hela cells 6 h after incubation with free MTX or with PEI/pDNA/MTX complexes, at different time points. Each data point represents the mean ± SD (n = 3) and were analyzed by two-way ANOVA with the Bonferroni test. A statistically significant difference was noticed for PEI/pDNA/MTX between T8 and the other groups (**** *p* < 0.0001). Significant levels of MTX were also observed for T12 (**** *p* < 0.0001). Additionally, the difference between MTX and PEI/pDNA/MTX is statistically significant for all time points (#### *p* < 0.0001). (**b**) Circadian oscillations are statistically significant as analyzed with CircWave (*p* < 0.001 for both MTX and PEI/pDNA/MTX).

To reveal the influence of circadian rhythm on cellular uptake/internalization, the released MTX in the cytosol of HeLa cells after transfection mediated by PEI/pDNA/MTX complexes has been quantified, for the 6 time points considered. The obtained results are presented in Figure 3a. From the cell-associated MTX fluorescence quantification, it seems that both free drug and the developed vectors can be internalized into HeLa cells, however in a different extent. For this can largely contribute the affinity of this drug for folate receptors, as was already investigated and proved, by our team, in a recent work [25]. Free MTX is uptaked into cells in a very low extent when compared with the content of MTX found when the transfection was mediated by PEI/pDNA/MTX delivery system. In fact, considering a delivery vector for MTX loading, led to improved internalization of the drug into cancer cells. This situation is most probably related with the poor bioavailability and low aqueous solubility of the drug, fact that can be overcome by its encapsulation into a drug carrier system [31,32]. In line with this, higher levels of MTX were quantified in HeLa cells when PEI/pDNA/MTX vectors were used. Comparing the different time points evaluated, significant levels of MTX were observed for T4, T8 and T12. Among those, the highest MTX amount was detected at T8, with statistically significant differences relatively to other time points (Table S1, available in Supplementary Materials). This finding suggested that vector´s cellular internalization, mediated by PEI/pDNA/MTX carrier, is more efficient at this time point. This assumption can be related with an easier vector´s cell entry, that speculatively, may be facilitated by the higher expression of folate receptors at this time point, in comparison to the other time points studied. Currently, and to clarify this premise, our investigation relies on the evaluation of this specific issue, but unfortunately, results cannot be anticipated at this stage. 

Furthermore, the circadian oscillation patterns of MTX quantification were evaluated and CircWave analysis can be consulted in Figure 3b. Interestingly, the MTX quantification exhibited a circadian pattern in both free MTX (*p* < 0.05) and when the transfection was mediated by PEI/pDNA/MTX delivery system (*p* < 0.05). In addition, peak time for the different situations (depicted as center of gravity (CG) was very similar, around T8 (Table S2 presented in Supplementary Materials). Thus, it may be concluded that free MTX or the developed vector internalization, are both circadian clock dependent. These results are in agreement with previous research where the circadian rhythmicity in MTX pharmacokinetics were demonstrated in animals and humans [33]. Notably, the internalization mediated by PEI/pDNA/MTX vector does not affect the peak of the circadian oscillation, when compared to the administration of the drug alone, considering the involvement of delivery vectors a potential strategy as cancer chronotherapy.

The role of circadian clock on tumorigenesis is currently well recognized. The disruption of circadian rhythm strongly influences tumorigenesis and facilitates the establishments of cancer hallmarks. In fact, it is possible that the transformation of normal to cancer cells might be promoted by the disruption of the circadian control. In addition, it was also described that anticancer chemotherapies are partly responsible for the escape of circadian control [34,35]. Contrary, oncogenic processes directly weaken circadian rhythms [34].

On the other hand, preliminary work on this field, revealed that controlled delivery systems were already tested to support timing drug delivery. In this sense, a three layered semipermeable polymeric formulation, the use of hydrogels, or more recently paclitaxel-based polymeric nanoparticles were examined for chrono-modulated drug delivery [27,36,37]. Thus, considering our and previous results, we deduce that the circadian clock functions as a sentinel, timing of MTX internalization. Curiously, the circadian system capacity to “sense” the cellular environment, adapted the response to the MTX and MTX-loaded vector and the synchronization was maintained. Overall, the therapy alone or combined with the delivery system does not disrupt the normal function of the circadian clock and consequently might not promote cancer initiation.

### 3.3. Fluorescence Microscopy Evaluation

**Figure 4:**
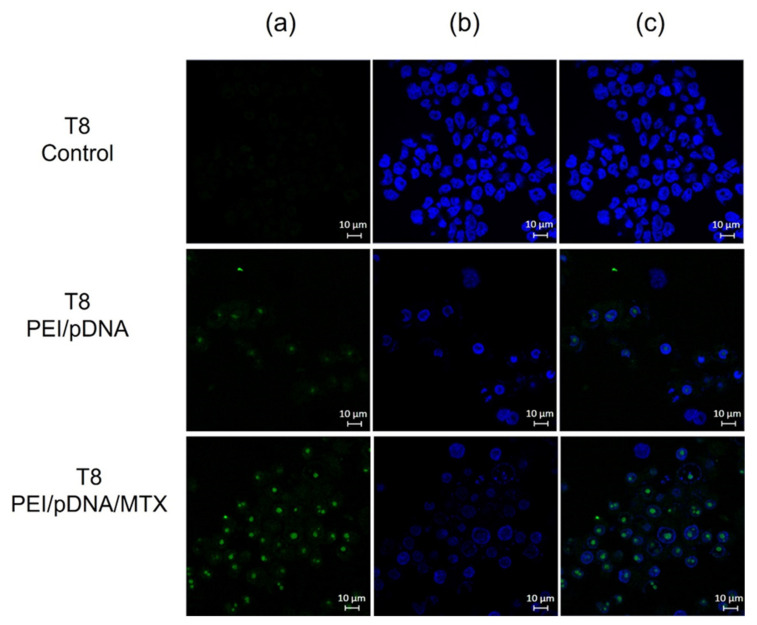
Fluorescence microscopy study at T8: a cell live imaging experiment illustrating PEI/pDNA/MTX nanoparticles uptake/internalization in HeLa cells, and nucleus co-localization. (**a**) DAPI labeled nucleus; (**b**) pDNA green stained with FITC; (**c**) Merged image. Scale bar = 10 μm.

To further evaluate the effectiveness of PEI/pDNA/MTX vectors for cellular uptake into HeLa cells and pDNA delivery, live cell imaging experiments were conducted. The study, performed for T8 is presented in Figure 4. Non-transfected cells were used as control. The pDNA is green labelled by FITC (a), nuclei are stained blue (b) and the merged image (c) gives an indication of the transfection efficiency. Images from the transfection mediated by PEI/pDNA or PEI/pDNA/MTX carriers show the presence of stained pDNA into cancer cells (c). The co-localization analysis demonstrates the location of pDNA, mainly, into the cell nucleus. However, it should not be neglected the possibility of some nanosystems being located into the cytoplasmic compartment and perinuclear space. Similar results were obtained for the transfection performed at T12 (data not shown). This evident and successful cellular uptake of PEI/pDNA/MTX particles, at T8, predicts their ability to surpass both extracellular and intracellular barriers transporting pDNA, i.e the genetic material, to the cytoplasm, and possibly, followed by nuclear trafficking to the nuclear periphery. pDNA can then be directed to the nucleus by the cell endogenous import machinery, where p53 gene can be expressed [38].

**Figure 5:**
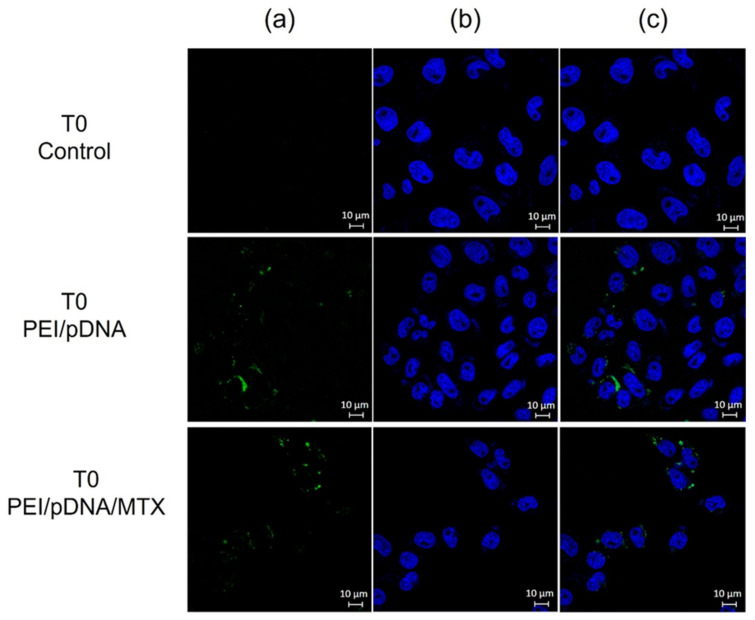
Cell live imaging experiment realized at T0 illustrating PEI/pDNA/MTX nanoparticles uptake/internalization in HeLa cells, and nucleus co-localization. (**a**) DAPI labeled nucleus; (**b**) pDNA green stained with FITC; (**c**) Merged image. Scale bar = 10 μm.

To highlight the differences between the time points considered, in contrast, Figure 5 shows the low transfection efficiency of both PEI/pDNA and PEI/pDNA/MTX based systems at T0. These images, indicate the poor cellular internalization of the carriers, at T0, into HeLa cells, evidenced by the low FITC-pDNA fluorescence (Figure 5b). Moreover, the few uptaked nanoparticles seem to be located in the cytoplasm of cancer cells (Figure 5c). This live cell imaging study at T0 is consistent with low cellular uptake/internalization and, therefore, inefficient transfection at this timepoint.

### 3.4. The Effect of Circadian Rhythms on Protein Expression: Quantification of p53 Expression

**Figure 6:**
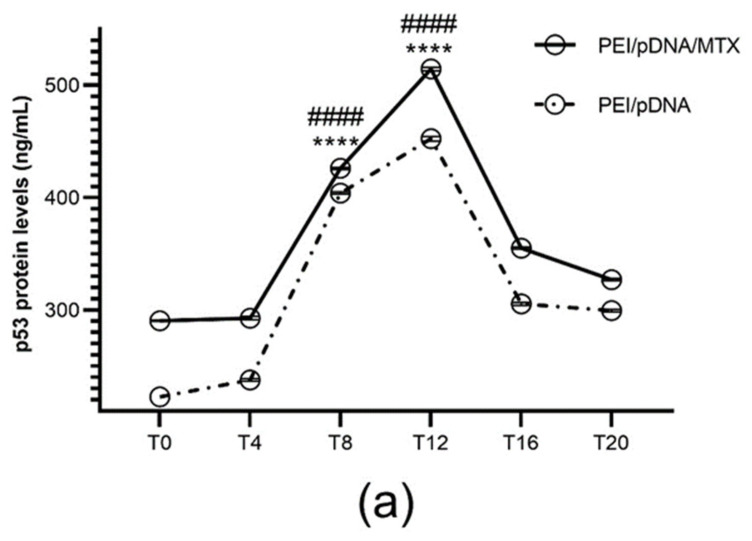

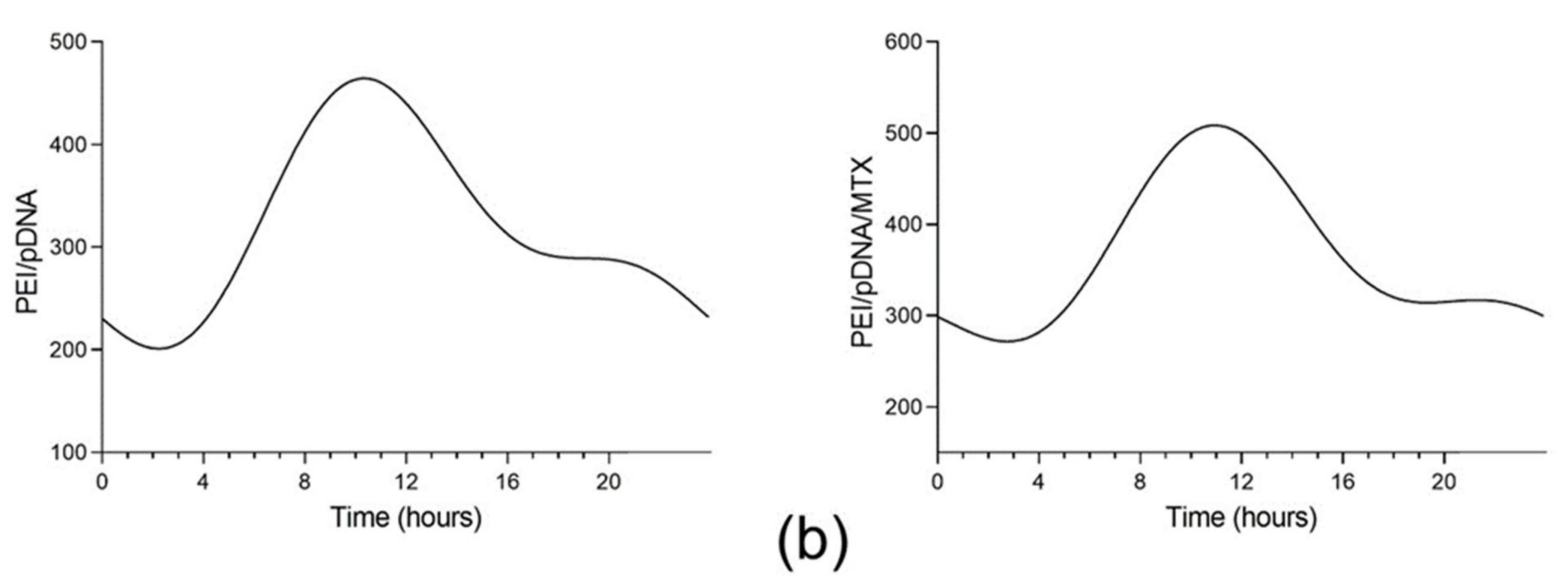
Quantification of p53 protein levels (ng/mL) for each time point. (**a**) p53 protein levels have been determined 48 h after transfection mediated by PEI/pDNA and PEI/pDNA/MTX nanoparticles by using p53 pan ELISA kit. Each data point represents the mean ± SD (n = 3) and were analyzed by two-way ANOVA with the Bonferroni test. At time T8 and T12, substantial levels of protein were found in Hela cells, in comparison to other time points (**** *p * < 0.0001). Additionally, the difference between PEI/pDNA and PEI/pDNA/MTX is statistically significant for T8 and T12 (#### *p*< 0.0001). (**b**) Circadian oscillations are statistically significant as analyzed with CircWave (*p* = 0 for both PEI/pDNA and PEI/pDNA/MTX).

After observing the ability of PEI/pDNA/MTX delivery system for cellular uptake, p53 protein expression was monitored for each time point considered. Non-transfected and transfected cells with PEI/pDNA vectors were used as control. p53 was quantified by using the ELISA immunoassay, as described above, and the results are presented in Figure 6a. The presence of protein was not detected in non-transfected cells (data not shown). Contrary, both carrier systems, with and without loaded MTX, were able to transfect cancer cells, promote gene expression and, ultimately, synthesize p53 protein in all the time points considered. Interestingly, differences were observed in p53 content levels. The presence of MTX into the vectors led to a higher amount of p53 expression and this increment was more pronounced for certain time points, namely T8 and T12 (Table S3, available in Supplementary Materials). CircWave graphs in Figure 6b demonstrated a rhythmic expression of p53 in both carriers. Again, the circadian analysis revealed a similar peak in the amount of p53 in both PEI/pDNA and PEI/pDNA/MTX vectors (~T12; Table S4, in Supplementary Materials). This fact correlates well with the above results concerning the higher capacity of the vectors to internalize into HeLa cells when transfection, mediated by PEI/pDNA/MTX nanoparticles, was realized at these particular time points. The performance of the studied delivery system seemed to be higher at specific times, what certainly improve a set of mechanisms such as folate receptor recognition, cellular internalization and gene expression resulting in high protein production. Therefore, a pronounced therapeutic effect can be expected at these time points.

### 3.5. The Influence of Circadian Rhythms in Different Cell Lines: Cancer Versus Non-Cancer Cells

To test the effect of circadian rhythms on other cell lines, C33A and fibroblast cells were used. This study allows to understand if circadian rhythms also influence nanoparticles uptake in these cells and may unravel a different circadian pattern between cancer and non-cancer cells. Following this, cellular internalization of MTX and p53 expression were quantified in both C33A and fibroblast cells, as described above. 

**Figure 7:**
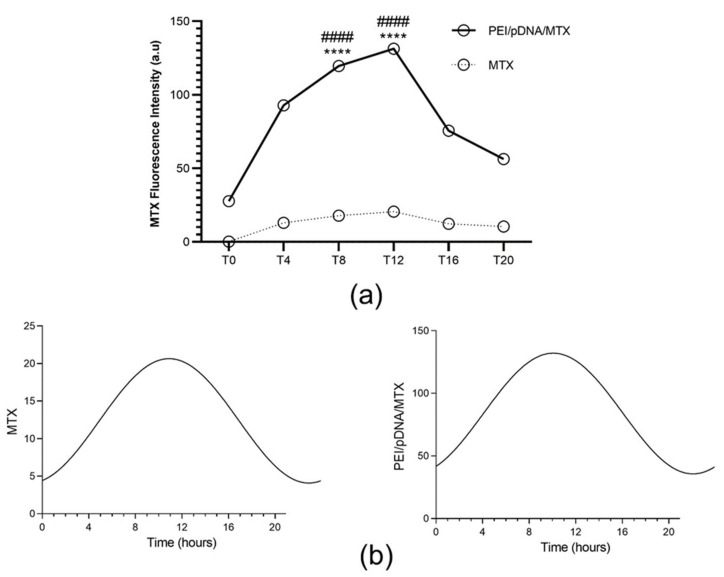
Quantification of cell-associated MTX fluorescence for each time point. (**a**) The fluorescence intensity of MTX was measured in C33A cells 6 h after incubation with free MTX or with PEI/pDNA/MTX complexes, at different time points. Each data point represents the mean ± SD (n = 3) and were analyzed by two-way ANOVA with the Bonferroni test. A statistically significant difference was noticed for PEI/pDNA/MTX between T12 and the other groups (**** *p* < 0.0001). Significant levels of MTX were also observed for T8 (**** *p* < 0.0001). Additionally, the difference between MTX and PEI/pDNA/MTX is statistically significant for all the time points (#### *p* < 0.0001). (**b**) Circadian oscillations are statistically significant as analyzed with CircWave (*p* < 0.001 for both MTX and PEI/pDNA/MTX).

Regarding MTX quantification, C33A cells also showed the capability to internalize both free MTX and the polyplexes in a circadian manner (Figure 7a). Similar to the profile observed before for HeLa cells, significant levels of MTX were found for T8 and T12, for transfection mediated by PEI/pDNA/MTX complexes, with higher levels detected at T12. The full statistical analysis can be consulted in Supplementary Materials, Table S5. The rhythmicity of MTX release in cytoplasm was also proved by CircWave analysis (Figure 7b). Once again, a similar pattern in MTX quantification is observed for both free MTX and transfection mediated by PEI/pDNA/MTX carriers, with a peak time around T10 (*p* < 0.0001) (Table S6, available in Supplementary Materials). Therefore, data seemed to indicate that MTX internalization (alone or incorporated in the complexes) is also coordinated by the circadian machinery in C33A cancer cell line.

**Figure 8:**
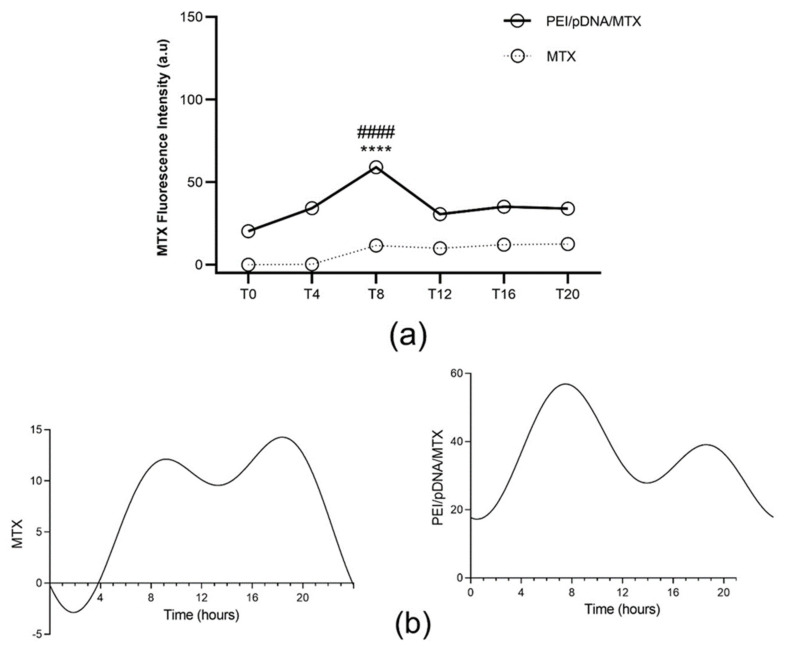
Quantification of cell-associated MTX fluorescence for each time point. (**a**) The fluorescence intensity of MTX was measured in fibroblast cells 6 h after incubation with free MTX or with PEI/pDNA/MTX complexes, at different time points. Each data point represents the mean ± SD (n = 3) and were analyzed by two-way ANOVA with the Bonferroni test. A statistically significant difference was noticed for PEI/pDNA/MTX between T8 and the other groups (**** *p* < 0.0001). Additionally, the difference between MTX and PEI/pDNA/MTX is statistically significant for all the time points (#### *p* < 0.0001). (**b**) Circadian oscillations are statistically significant as analyzed with CircWave (*p* < 0.001 for both MTX and PEI/pDNA/MTX).

Concerning the study performed on fibroblast cells, MTX was also detected in the cytosol of fibroblasts, although at low extent (Figure 8a). This was an expected result, as it had been previously observed that the presence of MTX into the developed complexes enhances cancer cells targeting ability. This favors cellular uptake through folate receptors, as these receptors are overexpressed in cancer cells [25]. Interesting to notice, was the fact that the amount of MTX released in the cytosol was also circadian dependent. In this case, a higher level of MTX was found at T8 for transfection mediated by PEI/pDNA/MTX, with statistically significant differences relatively to other time points (Table S7 in Supplementary Materials). In both cases, CircWave analyses proves the rhythmicity of MTX release in the cytosol of fibroblasts (Figure 8b), but with different time peaks for internalized MTX alone (center of gravity around T14) and complexed MTX (center of gravity around T9), as can be analyzed in Table S8 (SM). Fibroblast cells have been proved to function as independent circadian oscillators [39], so it is reasonable to consider that the internalization of nanocomplexes in these cells can be influenced by circadian system.

**Figure 9:**
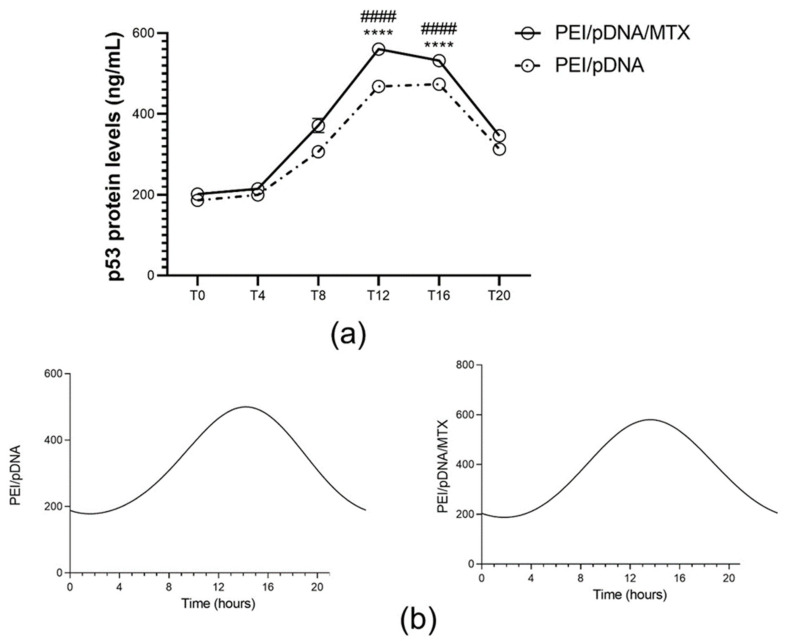
Quantification of p53 protein levels (ng/mL) for each time point. (**a**) p53 protein levels have been determined 48 h after transfection mediated by PEI/pDNA and PEI/pDNA/MTX nanoparticles by using the p53 pan ELISA kit. Each data point represents the mean ± SD (n = 3) and were analyzed by two-way ANOVA with the Bonferroni test. At time T12 and T16, substantial levels of protein were found in C33A cells, in comparison to other time points (**** *p* < 0.0001). Additionally, the difference between PEI/pDNA and PEI/pDNA/MTX is statistically significant for T12 and T16 (#### *p*< 0.0001). (**b**) Circadian oscillations are statistically significant as analyzed with CircWave (*p* = 0 for both PEI/pDNA and PEI/pDNA/MTX).

The p53 content after transfection was also evaluated and it was detected in both cell lines, however at different extents. In C33A, high levels of protein were detected, particularly at T12 (Figure 9a), with statistically significant differences comparatively to other time points (Table S9, SM). In addition, the values obtained were similar to those detected on Hela cells. In fact, these cell lines seem to have a similar behavior concerning the uptake and internalization of PEI/pDNA/MTX nanosystems. Furthermore, CircWave analyses in Figure 9b demonstrated a rhythmic expression of p53 for C33A cells transfection mediated by PEI/pDNA and PEI/pDNA/MTX carrier, with a center of gravity around T14 for both cases (Table S10, available in Supplementary Materials).

**Figure 10:**
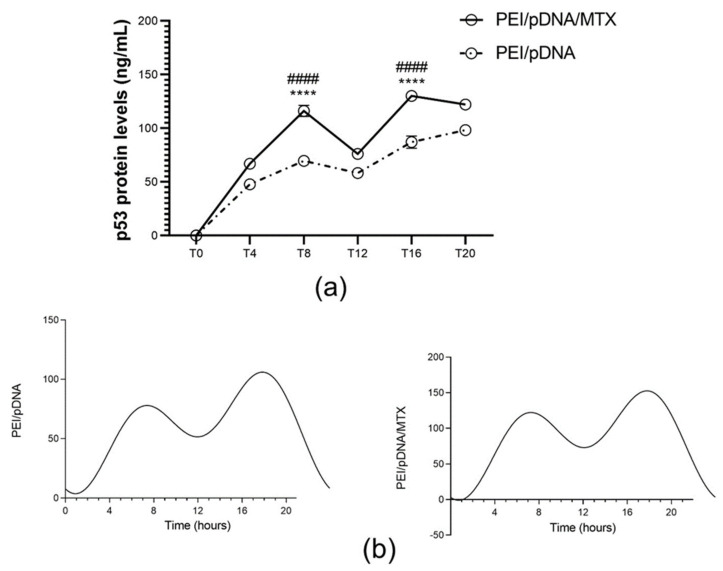
Quantification of p53 protein levels (ng/mL) for each time point. (**a**) p53 protein levels have been determined 48 h after transfection mediated by PEI/pDNA and PEI/pDNA/MTX nanoparticles by using the p53 pan ELISA kit. Each data point represents the mean ± SD (n =3) and were analyzed by two-way ANOVA with the Bonferroni test. At time T8 and T16, substantial levels of protein were found in fibroblasts, in comparison to other time points (**** *p* < 0.0001). Additionally, the difference between PEI/pDNA and PEI/pDNA/MTX is statistically significant for T8 and T16 (#### *p*< 0.0001). (**b**) Circadian oscillations are statistically significant as analyzed with CircWave (*p* < 0.0001 for both PEI/pDNA and PEI/pDNA/MTX).

In fibroblasts, two non-consecutive peaks of p53 were observed, one at T8 and other at T16 (Figure 10a) for transfection mediated by PEI/pDNA/MTX, and T8 and T20 for transfection with PEI/pDNA, with significant differences between the other time points (Table S11, SM). In both, the analysis of CircWave shows rhythmicity in p53 quantity and a center of gravity of about T14 (Table S12, available in Supplementary Materials).

**Figure 11:**
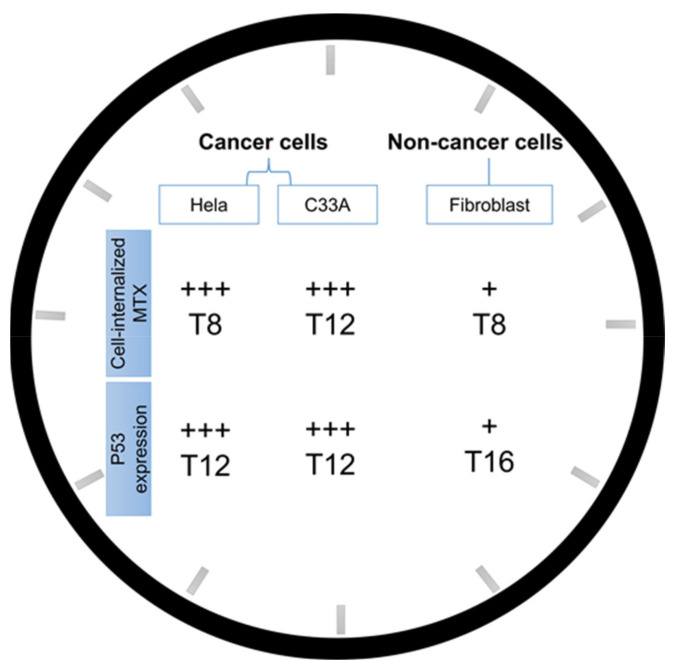
Schematic illustration summarizing the main findings of the current investigation on the influence of circadian rhythm on the performance of PEI/pDNA/MTX nanoparticles. Phenomena such as cellular uptake/internalization, cancer-cells targeting or protein expression has been monitored by measuring cell-internalized MTX and p53 protein quantification in HeLa, C33A and fibroblast cells.

This pattern was slightly different from the observed trend displayed by cancer cells (HeLa and C33A), nonetheless, a high amount of MTX in cytosol corresponds to greater amount of protein content. The comparative study between HeLa and C33A and fibroblast cells allowed to confirm once more the ability of the formed nanosystems to target cancer cells. Moreover, this comparative study on cancer versus non-cancer cells, seemed to demonstrate that, in both cases, circadian clock plays an important role in phenomena such as nanoparticles uptake and internalization, therefore, influencing the success of cellular transfection. Figure 11 summarizes the findings of the current study, evidencing the different profile achieved for cancer and non-cancer cells. Collectively, our results are highlighting the importance of circadian rhythms studies concerning the subject of chronotherapy.

## 4. Conclusions

Pursuing the goal of the development of novel, advanced and personalized cancer therapies towards improved therapeutic outcomes, in this work and for the first time, the effect of circadian rhythm in the performance of a drug/gene co-delivery system has been studied. Our investigation was mainly based on the analysis of transfection, in HeLa cells, mediated by PEI/pDNA/MTX complexes at different time points. The study included measurements of cell-associated MTX fluorescence, fluorescence confocal microscopy experiments and p53 protein quantification. The obtained results demonstrated higher performance of PEI/pDNA/MTX delivery vectors at specific time points. This work revealed that the ability of cellular uptake, drug/gene delivery, gene expression, and consequently, protein production seemed to be circadian regulated. Furthermore, the study in other cell lines, cancer and non-cancer cells, proved the influence of circadian rhythms in the internalization process of PEI/pDNA/MTX nanoparticles. The data seemed to indicate an optimum time for drug/gene administration stimulating additional investigation on this topic, as it may provide a tool to increase cancer therapeutic effect. This report unraveled important aspects regarding the influence of circadian clock on the “modus operandi” of a polycation co-delivery system for cancer therapy, instigating further research aiming the standard implementation of cancer chronotherapy. We are however aware that the presented work is preliminary, and more studies will be needed to fully clarify the impact of circadian timing system on cancer therapy. The identification of potential circadian rhythm targets involved or associated with cancer can result in optimal/specific drug chrono-delivery. Moreover, future investigation should also include the study of circadian biomarkers, that will bring understanding on the inter- and intra-individual variances allowing for progresses towards personalized cancer chronotherapy.

## Data Availability

The data presented in this study are available in main document and Supplementary Materials.

## Supplementary Materials

The following are available online at https://www.mdpi.com/article/10.3390/polym14040681/s1, Figure S1: CircWave analysis of bmal1 rhythmicity after Hela cells synchronization, Table S1: Statistical analysis of Bonferroni’s multiple comparisons test for the quantification of cell-associated MTX fluorescence on HeLa cells, Table S2: Significance (*p*-value) and center of gravity values for the quantification of cell-internalized MTX as determined by CircWave analysis, Table S3: Statistical analysis of Bonferroni’s multiple comparisons test for quantification of p53 protein levels between time points on Hela cells, Table S4: Significance (*p*-value) and center of gravity values for the quantification of p53 protein levels as determined by CircWave analysis on Hela cells, Table S5: Statistical analysis of Bonferroni’s multiple comparisons test for the quantification of cell-associated MTX fluorescence on C33A cells, Table S6: Significance (*p*-value) and center of gravity values for the quantification of cell-internalized MTX as determined by CircWave analysis, Table S7: Statistical analysis of Bonferroni’s multiple comparisons test for the quantification of cell-associated MTX fluorescence on fibroblasts, Table S8: Significance (*p*-value) and center of gravity values for the quantification of cell-internalized MTX as determined by CircWave analysis on fibroblasts, Table S9: Statistical analysis of Bonferroni’s multiple comparisons test for the quantification of p53 protein levels between time points on C33A cells, Table S10: Significance (*p*-value) and center of gravity values for the quantification of p53 protein levels as determined by CircWave analysis on C33A cells. Table S11: Statistical analysis of Bonferroni’s multiple comparisons test for the quantification of p53 protein levels between time points on fibroblasts, Table S12: Significance (*p*-value) and center of gravity values for the quantification of p53 protein levels as determined by CircWave analysis on fibroblast cells.
