# Supplementary material for: The Influence of Circadian Rhythm on Cancer Cells Targeting and Transfection Efficiency of a Polycation-Drug/Gene Delivery Vector
Source: Polymers (Basel). 2022 Feb 10;14(4):681. doi: 10.3390/polym14040681 (PMC8875434; doi:10.3390/polym14040681)
Supplement: Supplementary file 1 [file polymers-14-00681-s001.zip › polymers-1564419-supplementary.pdf]

Supplementary Materials

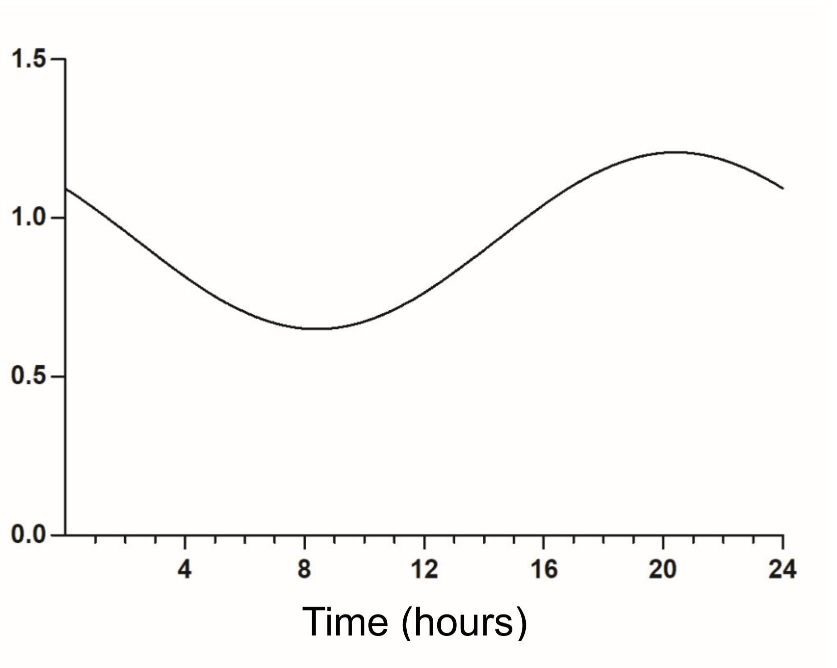

Figure S1. CircWave analysis of bmal1 rhythmicity after synchronization of HeLa cells.

On the 3th day of culture, cells were synchronized with 0.1 µM of dexamethasone for 2 h. After synchronization, the medium was replaced by fresh, dexamethasone free medium and cells were collected every 4 h through a cycle of 24 h. Relative mRNA expression levels were determined via qPCR, normalized to Cyclophilin A and cell synchronization was further confirmed by bmal1 expression analysis by CircWave (p-value= 0.026; center of gravity= 23.38).

Table S1. Statistical analysis of Bonferroni's multiple comparisons test for the quantification of cell-associated MTX fluorescence on Hela cells.

	**P-value**	
	MTX	PEI/MTX/pDNA
T0 vs. T4	0,0044	<0,0001
T0 vs. T8	<0,0001	<0,0001
T0 vs. T12	0,0194	<0,0001
T0 vs. T16	0,2468	<0,0001
T0 vs. T20	0,2607	0,0102
T4 vs. T8	0,0310	<0,0001
T4 vs. T12	>0,9999	>0,9999
T4 vs. T16	>0,9999	<0,0001

T4 vs. T20	>0,9999	<0,0001
T8 vs. T12	0,0071	<0,0001
T8 vs. T16	0,0005	<0,0001
T8 vs. T20	0,0004	<0,0001
T12 vs. T16	>0,9999	<0,0001
T12 vs. T20	>0,9999	<0,0001
T16 vs. T20	>0,9999	0,0055

	**P-value**
	MTX vs. PEI/pDNA/MTX
T0	<0,0001
T4	<0,0001
T8	<0,0001
T12	<0,0001
T16	<0,0001
T20	<0,0001

MTX, methotrexate; PEI, polyethylenimine; pDNA, p53 encoded plasmid DNA.

Table S2. Significance (p-value) and center of gravity values for the quantification of cell-internalized MTX as determined by CircWave analysis.

	**MTX**	**PEI/pDNA/MTX**
CG	8.82	8.48
P-value	4.4x10-5	2x10-6

CG, center of gravity.

Table S3. Statistical analysis of Bonferroni's multiple comparisons test for quantification of p53 protein levels between time points on Hela cells.

	**P-value**	
	PEI/pDNA	PEI/MTX/pDNA
T0 vs. T4	<0,0001	<0,0001
T0 vs. T8	<0,0001	<0,0001

T0 vs. T12	<0,0001	<0,0001
T0 vs. T16	<0,0001	<0,0001
T0 vs. T20	<0,0001	<0,0001
T4 vs. T8	<0,0001	<0,0001
T4 vs. T12	<0,0001	<0,0001
T4 vs. T16	<0,0001	<0,0001
T4 vs. T20	<0,0001	<0,0001
T8 vs. T12	<0,0001	<0,0001
T8 vs. T16	<0,0001	<0,0001
T8 vs. T20	<0,0001	<0,0001
T12 vs. T16	<0,0001	<0,0001
T12 vs. T20	<0,0001	<0,0001
T16 vs. T20	0,0003	<0,0001

P-value

PEI/pDNA vs. PEI/pDNA/MTX

T0	<0,0001
T4	<0,0001
T8	<0,0001
T12	<0,0001
T16	<0,0001
T20	<0,0001

Table S4. Significance (p-value) and center of gravity values for the quantification of p53 protein levels as determined by CircWave analysis on Hela cells.

	**PEI/pDNA**	**PEI/pDNA/MTX**
CG	10.73	10.63
P-value	0.0029	0.000201

Table S5. Statistical analysis of Bonferroni's multiple comparisons test for the quantification of cell-associated MTX fluorescence on C33A cells.

	**P-value**	
	MTX	PEI/MTX/pDNA
T0 vs. T4	<0,0001	<0,0001
T0 vs. T8	<0,0001	<0,0001
T0 vs. T12	<0,0001	<0,0001
T0 vs. T16	<0,0001	<0,0001
T0 vs. T20	<0,0001	<0,0001
T4 vs. T8	<0,0001	<0,0001
T4 vs. T12	<0,0001	<0,0001
T4 vs. T16	>0,9999	<0,0001
T4 vs. T20	0,0053	<0,0001
T8 vs. T12	0,0026	<0,0001
T8 vs. T16	<0,0001	<0,0001
T8 vs. T20	<0,0001	<0,0001
T12 vs. T16	<0,0001	<0,0001
T12 vs. T20	<0,0001	<0,0001
T16 vs. T20	0,0837	<0,0001

	**P-value**
	MTX vs. PEI/pDNA/MTX
T0	<0,0001
T4	<0,0001
T8	<0,0001
T12	<0,0001
T16	<0,0001
T20	<0,0001

Table S6. Significance (p-value) and center of gravity values for the quantification of cell-internalized MTX as determined by CircWave analysis on C33A cells.

	**MTX**	**PEI/pDNA/MTX**
CG	10.91	10.08

P-value	3x10-6	0

Table S7. Statistical analysis of Bonferroni's multiple comparisons test for the quantification of cell-associated MTX fluorescence on fibroblast cells.

	P-value	
	MTX	PEI/MTX/pDNA
T0 vs. T4	>0,9999	<0,0001
T0 vs. T8	<0,0001	<0,0001
T0 vs. T12	<0,0001	<0,0001
T0 vs. T16	<0,0001	<0,0001
T0 vs. T20	<0,0001	<0,0001
T4 vs. T8	<0,0001	<0,0001
T4 vs. T12	<0,0001	<0,0001
T4 vs. T16	<0,0001	0,0809
T4 vs. T20	<0,0001	>0,9999
T8 vs. T12	<0,0001	<0,0001
T8 vs. T16	0,5042	<0,0001
T8 vs. T20	0,0371	<0,0001
T12 vs. T16	<0,0001	<0,0001
T12 vs. T20	<0,0001	<0,0001
T16 vs. T20	>0,9999	0,0082

	P-value
	MTX vs. PEI/pDNA/MTX
T0	<0,0001
T4	<0,0001
T8	<0,0001
T12	<0,0001
T16	<0,0001
T20	<0,0001

Table S8. Significance (p-value) and center of gravity values for the quantification of cell-internalized MTX as determined by CircWave analysis on fibroblasts.

	**MTX**	**PEI/pDNA/MTX**
CG	14.38	9.2

P-value	0	0

Table S9. Statistical analysis of Bonferroni's multiple comparisons test for the quantification of p53 protein levels between time points on C33A cells.

	P-value	
	PEI/pDNA	PEI/MTX/pDNA
T0 vs. T4	0,1172	0,1717
T0 vs. T8	<0,0001	<0,0001
T0 vs. T12	<0,0001	<0,0001
T0 vs. T16	<0,0001	<0,0001
T0 vs. T20	<0,0001	<0,0001
T4 vs. T8	<0,0001	<0,0001
T4 vs. T12	<0,0001	<0,0001
T4 vs. T16	<0,0001	<0,0001
T4 vs. T20	<0,0001	<0,0001
T8 vs. T12	<0,0001	<0,0001
T8 vs. T16	<0,0001	<0,0001
T8 vs. T20	>0,9999	0,0002
T12 vs. T16	>0,9999	<0,0001
T12 vs. T20	<0,0001	<0,0001
T16 vs. T20	<0,0001	<0,0001

	P-value
	PEI/pDNA vs. PEI/pDNA/MTX
T0	0,0202
T4	0,0301
T8	<0,0001
T12	<0,0001
T16	<0,0001
T20	<0,0001

Table S10. Significance (p-value) and center of gravity values for the quantification of p53 protein levels as determined by CircWave analysis on C33A cells.

	**PEI/pDNA**	**PEI/pDNA/MTX**
CG	14.02	13.7
P-value	0	0

Table S11. Statistical analysis of Bonferroni's multiple comparisons test for the quantification of p53 protein levels between time points on fibroblasts.

	P-value	
	PEI/pDNA	PEI/MTX/pDNA
T0 vs. T4	<0,0001	<0,0001
T0 vs. T8	<0,0001	<0,0001
T0 vs. T12	<0,0001	<0,0001
T0 vs. T16	<0,0001	<0,0001
T0 vs. T20	<0,0001	<0,0001
T4 vs. T8	<0,0001	<0,0001
T4 vs. T12	0,0179	0,0562
T4 vs. T16	<0,0001	<0,0001
T4 vs. T20	<0,0001	<0,0001
T8 vs. T12	0,0100	<0,0001
T8 vs. T16	<0,0001	0,0009
T8 vs. T20	<0,0001	0,7714
T12 vs. T16	<0,0001	<0,0001
T12 vs. T20	<0,0001	<0,0001
T16 vs. T20	0,0091	0,1542

	P-value
	PEI/pDNA vs. PEI/pDNA/MTX
T0	>0,9999
T4	<0,0001
T8	<0,0001
T12	<0,0001
T16	<0,0001
T20	<0,0001

Table S12. Significance (p-value) and center of gravity values for the quantification of p53 protein levels as determined by CircWave analysis on fibroblast cells.

	**PEI/pDNA**	**PEI/pDNA/MTX**
CG	14.56	13.98
P-value	2x10-6	0
